# Oceanic barnacles act as foundation species on plastic debris: implications for marine dispersal

**DOI:** 10.1038/srep19987

**Published:** 2016-01-27

**Authors:** Michael A. Gil, Joseph B. Pfaller

**Affiliations:** 1 Department of Biology, University of Florida, Gainesville, FL 32611-8525, USA; 2 Archie Carr Center for Sea Turtle Research, Department of Biology, University of Florida, Gainesville, FL 32611, USA; 3 Caretta Research Project, Savannah, GA 31412, USA

## Abstract

Plastic has emerged as an abundant, stable substratum for oceanic dispersal of organisms via rafting. However, the ecological mechanisms underlying community diversity on plastic debris remain poorly understood. On a cruise from California to Hawai’i, we surveyed plastic debris, some likely originating from the 2011 Tōhoku tsunami, to examine the relationship between rafting community diversity and both habitat area and stalked barnacle (*Lepas* spp.) abundance. For sessile taxa richness, we observed an interaction in which the positive effect of debris area weakened the negative effect of barnacle cover. In contrast, for mobile taxa richness, including cohabiting species from opposite sides of the Pacific Ocean, barnacle abundance had a positive effect that was strongest at smaller debris sizes. These findings suggest that barnacles, through interactions with habitat area, have trait-dependent effects on other species, serving as both foundation species and competitors, mediating the diversity and dispersal potential of marine organisms on plastic debris.

Oceanic dispersal via rafting is an important mechanism by which organisms colonize new areas^1^. Such events, however, are often limited by the abundance, longevity and habitability of floating substrata, as well as the physiological tolerances of potential rafting organisms^2^,^3^. Natural rafts (e.g., wood, pumice and marine vegetation) are generally characterized by low or patchy abundance, limited longevity, and relatively high habitability, due to high surface rugosity, structural complexity, and biodegradability^3^. Historically, natural rafts provided the only opportunities for rafting dispersal, leading to infrequent or episodic oceanic dispersal events. However, over the last four decades, the abundance of buoyant plastic debris in the marine environment has increased at an alarming and accelerating rate^4^,^5^. In addition to its ever-rising abundance, the longevity of floating plastic debris can vastly exceed that of natural rafts^2^,^6^, allowing for greater dispersal potential for organisms that colonize plastic debris^5^,^7^. The emergence of plastic as an abundant and relatively stable substratum for rafting dispersal necessitates a better understanding of the factors that affect the diversity of rafting communities on plastic debris.

Recent data suggest that larger pieces of plastic debris support higher biological diversity^8^, consistent with classic species-area relationships inherent to island biogeography^9^,^10^. However, this relationship was confounded with debris complexity, as the most structurally complex debris (e.g., tangled clumps of rope) supported the highest diversity^11^. Plastic debris of all sizes often possess limited structural complexity and smooth, rigid surfaces (e.g., buoys, containers, balls, siding). These characteristics may limit the habitability of plastic debris for many species, given that a great variety of organisms require shelter to persist. Therefore, the extent to which a species-area relationship is robust across plastic debris remains poorly understood and additional biological mechanisms that may mediate this relationship remain unexplored.

The exposed environment of smooth plastic surfaces in the ocean may be analogous to natural environments, in which harsh conditions (e.g., high physical stress, nutrient limitation or predator pressure) promote positive species interactions (e.g., mutualism, facilitation) that, in turn, allow for a greater variety of organisms to persist^12^,^13^. It has been speculated that certain rafting organisms (e.g., stalked barnacles of the genus *Lepas*) promote colonization and persistence of other rafting taxa on natural debris, by providing additional structural complexity^14^. In this way, *Lepas* barnacles may serve as foundation species (*sensu* Dayton 1972)^15^, structuring the greater community by decreasing the severity of local conditions. Concordantly, such foundation species on plastic debris could alter these artificial rafts by increasing their habitability to a more diverse assemblage of rafting organisms, particularly small, mobile species that require structural refugia to persist on rafts. However, evidence for foundation species on floating debris has not been quantified nor has this been considered in the context of oceanic plastic debris. Therefore, we examined the effects and interactions of habitat area and *Lepas* barnacle abundance on rafting community diversity on structurally-limited plastic debris to better understand the role these different mechanisms may play in plastic-mediated dispersal of marine organisms.

## Results

**Figure 1:**
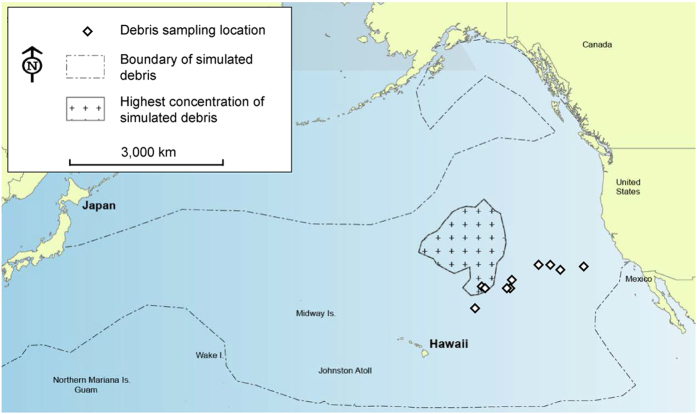
Plastic debris sampling locations (diamonds) across the North Pacific Subtropical Gyre from 9-31 October 2012. Plastic debris samples (N = 31) were collected from 14 locations within NOAA’s simulated debris field (containing 95% of 8,000 simulated pieces of debris) and fringing the highest concentration of simulated debris from the Tōhoku tsunami in Japan in March 2011 (hindcast simulation run from March 11, 2011 to November 5, 2012)^16^.

The plastic items collected in this study may have originated from an estimated 1.5 million tons of floating debris deposited into the Pacific Ocean during the Tōhoku tsunami, which took place in Japan on March 11, 2011 (estimates from a report by the Ministry of the Environment, Government of Japan). Though we cannot prove the origin of our sampled debris, our cruise track (from California to Hawai‘i) ran directly through the expected debris field and fringed the expected area of highest debris density, according to a hindcast simulation model run by the National Oceanic and Atmospheric Administration’s Office of Response and Restoration^16^ (Fig. 1). Furthermore, we sampled nine pieces of plastic debris that had confirmed Japanese markings and were widely distributed over most (9/14) of our sampling locations, including our easternmost (initial) and westernmost (final) sampling locations that bounded our study region (Fig. 1). Also, along our sampling trajectory we observed very large pieces of debris, including a refrigerator containing food in Japanese packaging and a capsized boat (Fig. S2D).

The plastic debris sampled were characterized by limited structural complexity, consisting of mostly spherical objects (e.g., buoys, toy balls), containers (e.g., bottles), and structural materials (e.g., pieces of insulation and siding), ranging in size from 16–67,749 cm^2^ (Tables S1 & S2). Despite the limited structural complexity that characterized our debris samples (Table S1), we found thriving communities of rafting organisms (Table S3), including taxa commonly found within coastal habitats, those not yet documented on oceanic rafts^3^, and, on one piece of debris, co-habiting taxa from opposite sides of the Pacific Ocean [Fig f2](Fig. 3). While our predictor variables (debris area and barnacle area for sessile taxa and debris area and number of barnacles for mobile taxa) were correlated (R^2^ = 0.59, p < 0.0001 and R^2^ = 0.49, p < 0.0001, respectively), the variance inflation factor was 2.54 and 2.04, for area + barnacle abundance models for sessile and mobile taxa, respectively, indicating little inflation in the variance of model coefficients due to co-linearity between predictor variables.

A summary of the results of our statistical modeling can be found in Table 1. Our model residuals verified that our data met assumptions of normality and homoscedasticity. Our models of main effects (area + barnacle abundance) better fit our data (sessile: AIC = 3.93; mobile: AIC = −31.32) than null models (sessile: AIC = 8.29; mobile: AIC = 5.37) and revealed contrasting patterns for sessile and mobile taxa richness. First, regarding the number of sessile taxa on debris, debris area had a significant positive effect (standardized partial regression coefficient [SPR] = 0.78, p = 0.0064; Figs 3A & S1A), while barnacle cover had a significant negative effect (SPR = −0.61, p = 0.027). However, regarding the number of mobile taxa on debris, debris area had an insignificant positive effect (SPR = 0.14, p = 0.31), while the number of barnacles had a significant and much greater positive effect (SPR = 0.75, p < 0.0001; Figs 3B & S1B) on mobile the richness of mobile taxa.

The interaction models (area*barnacles) provided the best fit to the data (sessile: AIC = 0.54; mobile: AIC = −36.46) and also exhibited contrasting patterns for sessile and mobile taxa richness. For sessile taxa richness, a positive interaction arose (area:barnacle cover = 0.095, p = 0.032) because the negative effect of barnacle cover weakened with greater habitat area, which had a consistent positive effect on the number of sessile taxa (Figs 3A & S1A). Conversely, for mobile taxa richness, a negative interaction arose (area:number of barnacles = −0.080, p = 0.014) because the positive effect of the number of barnacles weakened with greater habitat area (Figs 3B & S1B). Using open surface area in place of submerged surface area as a predictor variable did not qualitatively change our conclusions; for sessile taxa, the interaction model containing submerged surface area provided a better fit, and for mobile taxa, the interactions models containing either submerged surface area or open surface area provided equivalent fits to the data^17^ (Table S4).

## Discussion

Our data indicate that oceanic plastic debris with higher *Lepas* barnacle abundance support a greater number of mobile taxa, such as primarily coastal species, including cohabiting species from opposite sides of the Pacific Ocean (Fig. 3B). These results suggest that *Lepas* barnacles act as foundation species in rafting communities (*sensu* Dayton 1972)^15^, by providing complex structural habitat on otherwise structurally limited plastic debris. The structural habitat provided by *Lepas* barnacles could facilitate settlement of immigrating organisms, e.g., adults or larvae originating from potentially faraway coastlines^18^ or other rafts^19^, and/or enhance the survival of new and old resident organisms on plastic debris. However, the positive effect of barnacles on the number of mobile taxa decreased gradually with greater debris area (Fig. 3B), an interaction that could be the result of differences in the spatial configuration of barnacles and/or the strength of species interactions (e.g., facilitation, competition, predation) across levels of habitat availability^20^,^21^,^22^,^23^,^24^. Our data further show that the positive species-area relationship recently observed among plastic rafts^8^,^11^ could be inhibited by barnacles for sessile taxa, which may be outcompeted^25^,^26^ (Figs 3A & S1A). However, this inhibitory effect vanished on debris with greater available habitat area, which may alleviate competition among sessile taxa (*sensu* Ambuel and Temple 1983)^20^. These results suggest that the species-area relationship on plastic debris can be driven by two potentially interactive mechanisms, including enhanced colonization and persistence of sessile taxa with greater raft area (i.e., the classic biological mechanism^9^), as well as facilitation of mobile taxa (and potential inhibition of sessile taxa) with greater *Lepas* barnacle abundance. Our findings reveal new insight regarding the mechanisms underlying community diversity on oceanic rafts and further implicate plastic debris as a driver of transoceanic species dispersal.

**Figure 2 f2:**
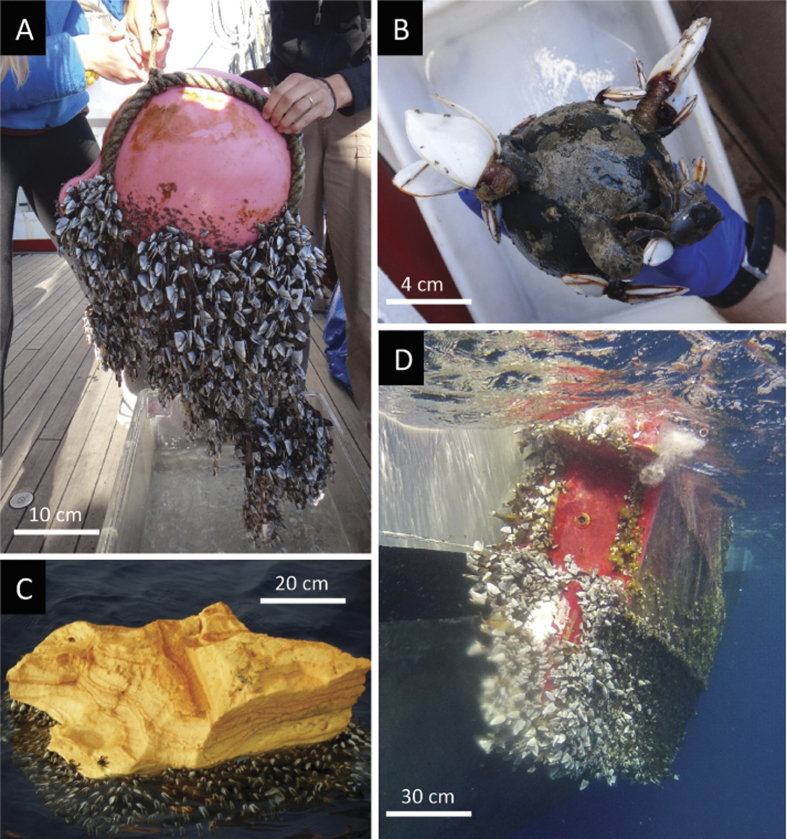
Plastic debris encountered on our expedition (October 2012), likely originating from the 2011 Tōhoku tsunami in Japan. Pictured are sampled buoys with Japanese writing (**A** & **B**), as well as sighted debris not brought on board, including a large chunk of foam (**C**) and a small capsized boat (**D**), all of which were covered in Lepas barnacles. Photo credits: Michael Gil (**A**, **B**), Patricia Keoughan (**C**), and Jon Waterman (**D**).

**Figure 3:**
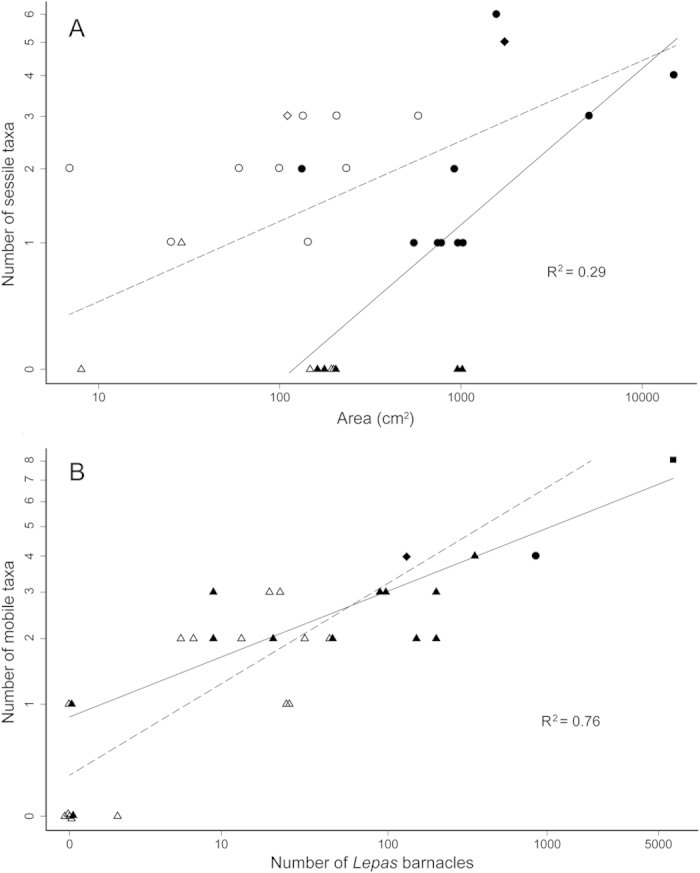
The relationship between (**A**) submerged surface area of plastic debris and the number of sessile taxa; and (**B**) the number of adult *Lepas* barnacles and the number of mobile taxa on the log-log scale. In each plot, data points represent each of the individual pieces of plastic debris sampled, which included: taxa primarily documented on oceanic rafts (a), taxa commonly documented in coastal habitats (b), coastal taxa not previously documented on oceanic rafts (c), and coastal taxa from the East Pacific Ocean co-occurring with coastal taxa from the West Pacific Ocean (d). The shape of each data point designates the type(s) of sessile (for panel **A**) or mobile (for panel **B**) taxa found on the debris piece: triangle, a; circle, a + b; diamond, a + b + c; or square, a + b + c + d. To visualize the interaction between predictor variables in each plot, we separated debris into two groups: those from the lower half (open points) or upper half (closed points) of the range of values of the second predictor variable (barnacle cover for A and submerged surface area for B). Lines represent predictions of the best-fit model (interaction model; sessile taxa: R^2^ = 0.29, p = 0.0068; mobile taxa: R^2^ = 0.76, p < 0.0001) for the average of either the low (dotted line) or high (solid line) values of the second predictor variable. Overlapping points (at 0 barnacles in B) were jittered for visual clarity.

Currently, it is estimated that 75.4% of the over 250,000 tons of plastic floating in the world’s oceans consists of debris >20 cm in diameter, of which 58.3% are derelict fishing buoys like many of our sampled debris^27^. The smooth, rigid surfaces of such numerous plastics (including those sampled in this study) provide little to no structural refugia for rafting organisms to remediate the mid-ocean surface’s harsh environmental conditions, including wave disturbance, limited food availability and predation by pelagic fishes^3^,^28^. However, *Lepas* barnacles readily recruit to and proliferate on smooth, rigid surfaces of plastic rafts (Figs. 2 and 3B), and in doing so appear to create structural niche space in a similar way as trees, reef corals, and marsh grasses^13^,^26^,^29^. Indeed, barnacles are well-known foundation species of rocky intertidal habitats, with acorn barnacles facilitating diversity in this similarly harsh environment^30^,^31^. Barnacles facilitating diversity on floating plastic debris or rocky shores coincides with Bertness and Callaway’s (1994) classic theoretical model, which postulates that biotic pressures (e.g., predation and competition) and abiotic stress (e.g., wave action) promote positive species interactions, including associational defenses and habitat amelioration, respectively^12^. Moreover, *Lepas* spp. have been notably sparse or absent within diverse rafting communities on structurally complex floating islands (e.g., tangled masses of rope)^11^, suggesting colonization by these barnacles and their facilitative relationship with other rafting taxa depends on substrate context.

Foundation species are highly valued because of their ability to facilitate species diversity in socioeconomically important ecosystems^29^,^32^,^33^. However, recent works have shown that foundation species in natural ecosystems can also facilitate the local abundances and diversity of invasive species^34^,^35^, which pose a leading threat to global biodiversity and can devastate ecosystem services upon which humans depend^36^,^37^. Though mechanistically distinct, our data suggest a similar phenomenon: *Lepas* barnacles, by facilitating diversity on highly mobile artificial plastic raft systems, could allow rafts to transport species across vast expanses of ocean to new locations (Fig. 1), including valuable coastal ecosystems^5^,^38^,^39^. Indeed, some of the debris we sampled likely originated >6,500 km away in Japan, including a Japanese buoy that supported a crab species from the West Pacific (*Glebocarcinus amphioetus*), in addition to species from the East Pacific (which could have arrived as larvae or originated from other debris; Fig. 3). In turn, plastic marine debris, particularly larger pieces and/or those with a higher abundance of foundation species, pose a rising global threat to coastal ecosystems via species invasions, as pollution increases due to both humans and natural disasters^40^. Our findings point to the need for further investigation of the ecological mechanisms that shape rafting communities on plastics and how these mechanisms may interact with both organism life histories (e.g., sessile vs. mobile, brooding vs. spawning, sexual vs. asexual) and properties of plastic debris itself (e.g., in addition to size: complexity, isolation, and duration at sea). Examining these relationships will not only improve our general understanding of the structure and maintenance of rafting communities, but it could provide insights (e.g., conditions that yield the highest diversity in rafting communities) that help remediate harmful effects of marine plastic pollution, such as species invasions.

## Materials and Methods

In October 2012, we sampled communities of organisms on floating plastic debris in the North Pacific Subtropical Gyre, during the Plastics @ SEA North Pacific Expedition aboard the *SSV Robert C. Seamans* (operated by Sea Education Association, Woods Hole, MA, USA). We sampled 31 plastic debris pieces from 14 locations that covered a distance of 2,300 km, beginning from 622 km off the coast of California to 1,158 km off the coast of Hawai’i (Fig. 1). Plastic debris pieces were haphazardly sampled from the ocean surface, based on daytime deck sightings, ship maneuverability, availability of personnel to process samples and our ability to bring the debris on board for analysis (i.e., pieces >1.5 m diameter could not be brought on board and were excluded from this study). Sampled debris pieces were quickly brought on board using dip nets and placed into tubs to minimize the loss of mobile taxa.

Once each piece of debris was brought aboard, it was visually inspected (using the naked eye) for rafting taxa ≥5 mm long, which were removed using hands and scraping tools (e.g., spatulas, knives, paint scrapers). Mobile taxa and sessile taxa with discrete individuals (e.g., anemones, *Lepas* barnacles) were counted, while the relative areal cover of all sessile fauna was estimated visually and then converted to absolute measures of surface area, using total surface area measurements for each piece of debris (described below). We considered *Lepas* barnacles of <1 cm test length to be juveniles and we recorded their areal coverage only, while barnacles larger than this size were considered adults and were counted discretely. When >100 adult *Lepas* barnacles were present on a piece of debris, we estimated the number of adult barnacles by first removing the barnacles from the debris and then breaking the removed barnacles up into smaller groups of similar volume and barnacle size distribution, which was quite uniform in many cases. We then counted the number of adult barnacles in a single group and multiplied this amount by the total number of groups that comprised the full sample of barnacles that we removed from the debris, making every effort to keep counts consistent and accurate. We photographed and catalogued each novel morphospecies encountered, identified by distinct morphological characteristics and with the assistance of species identification guides. After the cruise, voucher samples of taxa (preserved in 70% ethanol) were identified to the lowest possible taxonomic level (genus or species, in most cases), with the assistance of expert zoologists associated with the Florida Museum of Natural History. Voucher samples are now housed at the Florida Museum of Natural History.

The total surface area of each debris piece was calculated by the same person (precluding observer effects), using meter tape measurements and geometric equations that corresponded to the shape of the debris piece or components thereof (Tables S1 and S2). Because each debris piece was only partially submerged (Table S1) and submerged habitat is required by marine organisms, we noted the orientation at the water surface and the locations of water lines and biofilms for each piece of debris to determine the ‘submerged surface area’. We used this as our primary predictor variable for habitat area. In addition, we examined a second predictor variable that provided a measure of habitat area that was independent of barnacle effects, by subtracting barnacle cover from submerged surface area, yielding a measure of ‘open surface area’, available to non-barnacle sessile colonizers. Once all living material was removed, each plastic debris piece was soaked in bleach, stored in the ship’s cargo hold, and either recycled or donated upon completion of the cruise.

We used linear regression models run in the program R 3.1.1^41^ to analyze main effects and interactions of debris surface area and *Lepas* barnacle abundance on the number of both sessile and mobile taxa. We log-transformed (using a + 1 correction, when necessary) both our predictor variables, to improve coverage and visualization, and our response variables, to meet model assumptions of normality and homoscedasticity. We used the variance inflation factor (in the *car* package^42^) to assess the effect of co-linearity between our two predictor variables, for each of which we calculated standardized partial regression coefficients (i.e., beta weights) to quantify their relative importance in multiple regression models. We used the corrected Akaike Information Criterion (AICc) to compare the relative fit of linear models with (1) only an intercept (null model), (2) debris surface area as a predictor, (3) barnacle abundance as a predictor, (4) both debris surface area and barnacle abundance as predictors, and (5) the interaction between debris surface area and barnacle abundance. We considered models to be unequal in their fit to the data if they differed in AICc (ΔAICc) by 2 or more units^43^. Adjustments for sampling effort (e.g., rarefaction) were unnecessary in our dataset, because our sampling effort scaled with debris size (i.e., we surveyed the entire surface of each debris piece).

## Additional Information

**How to cite this article**: Gil, M. A. and Pfaller, J. B. Oceanic barnacles act as foundation species on plastic debris: implications for marine dispersal. *Sci. Rep.* **6**, 19987; doi: 10.1038/srep19987 (2016).

## Supplementary Material

Supplementary Information

## Figures and Tables

**Table 1:** Summary of statistical modeling results, comparing the estimated coefficients and relative fits (denoted by AICc; lower value = better fit to data) of linear regression models from the number of sessile taxa and the number of mobile taxa collected from sampled plastic debris.

Response	Model	Coefficients (p-values)	AICc
No. sessile taxa	null (intercept only)	NA	8.72
	submerged surface area (SSA)	0.11 (0.10)	8.31
	barnacle cover	−0.003 (0.94)	11.17
	SSA + barnacle cover†	SSA = 0.78 (0.0064); barnacles = −0.61 (0.027)	5.02
	SSA * barnacle cover	SSA:barnacles = 0.095 (0.032)	2.36
No. mobile taxa	null (intercept only)	NA	5.80
	SSA	0.23 (<0.0001)	−10.77
	no. of barnacles	0.21 (<0.0001)	−31.28
	SSA + no. of barnacles†	SSA = 0.14 (0.31); barnacles = 0.75 (<0.0001)	−29.78
	SSA * no. of barnacles	SSA:barnacles = −0.080 (0.014)	−34.06

^†^Standardized partial regression coefficients reported to compare relative effects of predictor variables.
