# Supplementary material for: Oceanic barnacles act as foundation species on plastic debris: implications for marine dispersal
Source: Sci Rep. 2016 Jan 27;6:19987. doi: 10.1038/srep19987 (PMC4728489; doi:10.1038/srep19987)
Supplement: Supplementary Information [file srep19987-s1.pdf]

Oceanic barnacles act as foundation species on plastic debris: implications for marine dispersal

Michael A. Gil[1]* and Joseph B. Pfaller[2,3]

Affiliations:

[1]Department of Biology, University of Florida, Gainesville, FL 32611-8525, USA

[2]Archie Carr Center for Sea Turtle Research, Department of Biology, University of Florida, Gainesville, FL 32611, USA

[3]Caretta Research Project, Savannah, GA 31412, USA

***Corresponding author:** M. A. Gil: m.gil@ufl.edu

Supplementary Material

Table S1: Summary of characteristics of each of the 31 plastic debris pieces sampled.

No.	Date	Latitude (N)	Longitude (W)	Total surface area (cm^2)	Submerged surface area (cm^2)	Description	Shape(s)
1	9-Oct-12	33° 29.6'	127° 42.9'	4,333	1,743	buoy with rope strand	sphere, cylinder
2	12-Oct-12	32° 59.3'	131° 38.1'	16	8	flat piece	rect. prism
3	14-Oct-12	33° 43.5'	133° 28.8'	2,247	764	buoy	sphere
4	14-Oct-12	33° 39.3'	133° 26.2'	191	29	toy ball	sphere
5	14-Oct-12	33° 39.3'	133° 26.2'	50	25	flat piece	rect. prism
6	14-Oct-12	33° 39.3'	133° 26.2'	21	7	bottle cap	cylinder
7	16-Oct-12	33° 34.3'	135° 26.6'	4,067	1,017	buoy	sphere
8	16-Oct-12	33° 33.8'	135° 25.9'	453	204	flat piece	rect. prism
9	16-Oct-12	33° 33.8'	135° 25.9'	536	198	flat piece	rect. prism

10	16-Oct-12	33° 33.8'	135° 25.9'	1,831	958	flat piece	rect. prism
11	16-Oct-12	33° 33.8'	135° 25.9'	639	147	bottle	cylinder
12	16-Oct-12	33° 33.8'	135° 25.9'	646	161	bottle	cylinder
13	22-Oct-12	31° 19.8'	140° 20.4'	1,839	552	buoy	sphere
14	22-Oct-12	31° 19.8'	140° 20.4'	2,141	964	buoy	sphere
15	22-Oct-12	31° 19.8'	140° 20.4'	382	176	flat piece	rect. prism
16	22-Oct-12	31° 19.8'	140° 20.4'	1528	752	flat piece	rect. prism
17	23-Oct-12	30° 12.0'	140° 41.8'	67,749	14,905	boat bumper	cylinder
18	24-Oct-12	30° 7.0'	141° 11.0'	584	193	flat piece	rect. prism
19	24-Oct-12	30° 7.0'	141° 11.0'	2,637	923	buoy	sphere
20	24-Oct-12	30° 7.0'	141° 11.0'	572	143	flat piece	rect. prism
21	24-Oct-12	30° 7.0'	141° 11.0'	199	60	buoy	sphere
22	24-Oct-12	30° 7.0'	141° 11.0'	920	580	flat piece	rect. prism
23	24-Oct-12	30° 9.3'	141° 12.7'	895	233	toy ball	sphere
24	24-Oct-12	30° 9.3'	141° 12.7'	604	133	bottle	cylinder
25	24-Oct-12	30° 3.4'	145° 3.4'	2,141	1,563	buoy	sphere
26	28-Oct-12	30° 24.0'	145° 45.8'	180	99	flat piece	rect. prism
27	28-Oct-12	30° 24.0'	145° 45.8'	220	110	flat piece	rect. prism
28	28-Oct-12	30° 24.0'	145° 45.8'	411	206	flat piece	rect. prism
29	28-Oct-12	30° 24.0'	145° 45.8'	269	135	flat piece	rect. prism
30	31-Oct-12	27° 0.0'	146° 46.9'	25,519	5,104	buoy	ellipsoid
31	31-Oct-12	26° 59.0'	146° 47.4'	2,038	1,019	buoy	sphere

Table S2: Information used to calculate debris surface area. The shape of the debris piece or the shape of components of the debris piece determined the metrics measured to calculate surface area from standard geometric equations.

**Shape**	**Metric(s) measured**	**Surface area calculation**	**Example debris**
Cylinder	width (= 2r)<br>& height	$2\pi + 2\pi rh$	base or neck of bottle, piece of rope, boat bumper
Ellipsoid	length (= 2a),<br>width (= 2b),<br>& height (= 2c)	$4\pi\left(\frac{ab^{1.6} + ac^{1.6} + bc^{1.6}}{3}\right)^{\frac{1}{1.6}}$	buoy
Rectangular prism	length, width, & height	$2(lw + lh + wh)$	piece of siding, insulation
Sphere	circumference<br>(= $2\pi r$)	$4\pi r^2$	buoy, toy ball

Table S3: Taxa sampled from floating plastic debris encountered between California and Hawaiʻi in October 2012 (see Fig. 1). Notation: EW = East and West Pacific; E = East Pacific only; W = West Pacific only; NB = neritic/benthic; OP = oceanic/pelagic; I = intertidal; Y = yes, has been found rafting on oceanic debris; N = no, has not been found rafting on oceanic debris. Numbers in parentheses indicate the number of morphospecies recorded per taxon, if it was greater than 1.

**Taxon**	**Phylum**	**Distribution**	**1° Habitat**	**Mobility**	**Rafting**[†]
**Chlorophyta (3)**	Chlorophyta	EW	NB	sessile	Y
**Rhodophyta (2)**	Rhodophyta	EW	NB	sessile	Y
**Hydrozoa**	Cnidaria	EW	OP/NB	sessile	Y
**Actiniidae**	Cnidaria	EW	NB	sessile	Y
**Metridiidae**	Cnidaria	EW	NB	sessile	N
Amphinome rostrata	Annelida	EW	OP/NB	mobile	Y
***Chaetopterus* sp.**	Annelida	EW	NB	sessile	N
***Parasabella* sp.**	Annelida	EW	NB	mobile	N
Hipponoe gaudichaudi	Annelida	EW	OP	mobile	Y
***Lepidonotus* sp.**	Annelida	EW	NB	mobile	N
***Mytilus* sp.**	Bivalvia	EW	NB	sessile	Y
Fiona pinnata	Mollusca	EW	OP	mobile	Y
Lottia pelta	Mollusca	E	NB	mobile	N
***Lepas* spp.**	Arthropoda	EW	OP	sessile	Y
Idotea metallica	Arthropoda	EW	OP	mobile	Y
Pentidotea wosnesenskii	Arthropoda	E	I	mobile	N
Glebocarcinus amphioetus	Arthropoda	W	NB	mobile	N
Plagusia squamosa	Arthropoda	EW	OP/NB	mobile	Y
Planes major	Arthropoda	EW	OP	mobile	Y

Planes marinus	Arthropoda	EW	OP	mobile	Y
***Membranipora* spp.**	Bryozoa	EW	OP/NB	sessile	Y
***Psenes* sp.**	Chordata	EW	OP	mobile	Y
**Pomacentridae**	Chordata	EW	NB	mobile	N

Notes. Information on current taxonomy, distributions and primary habitat was obtained from the World Register of Marine Speices (WoRMS, www.marinespecies.org) and the Marine Species Identification Portal (www.species-identification.org).
[†] Based on review by Thiel and Gutow (2005b)

Table S4: Summary of statistical modeling results from analyses including open surface area as the habitat area predictor variable. Provided are estimated coefficients and relative fits (denoted by cAIC; lower value = better fit to data) of linear regression models from the number of sessile taxa and the number of mobile taxa collected from sampled plastic debris.

**Response**	**Model**	**Coefficients (p-values)**	**AIC**
No. sessile taxa	open surface area (OSA)	0.12 (0.084)	7.92
	OSA + barnacle cover[†]	OSA = 0.47 (0.033); barnacle cover = -0.28 (0.20)	8.69
	OSA * barnacle cover	OSA:barnacles = 0.066 (0.23)	9.88
No. mobile taxa	open surface area (OSA)	0.16 (0.01)	1.54
	OSA + no. of barnacles[†]	OSA = 0.0086 (0.44); barnacles = 0.081 (< 0.0001)	-29.31
	OSA * no. of barnacles	OSA:barnacles = -0.093 (0.010)	-34.18

[†]Standardized partial regression coefficients reported to compare relative effects of predictor variables

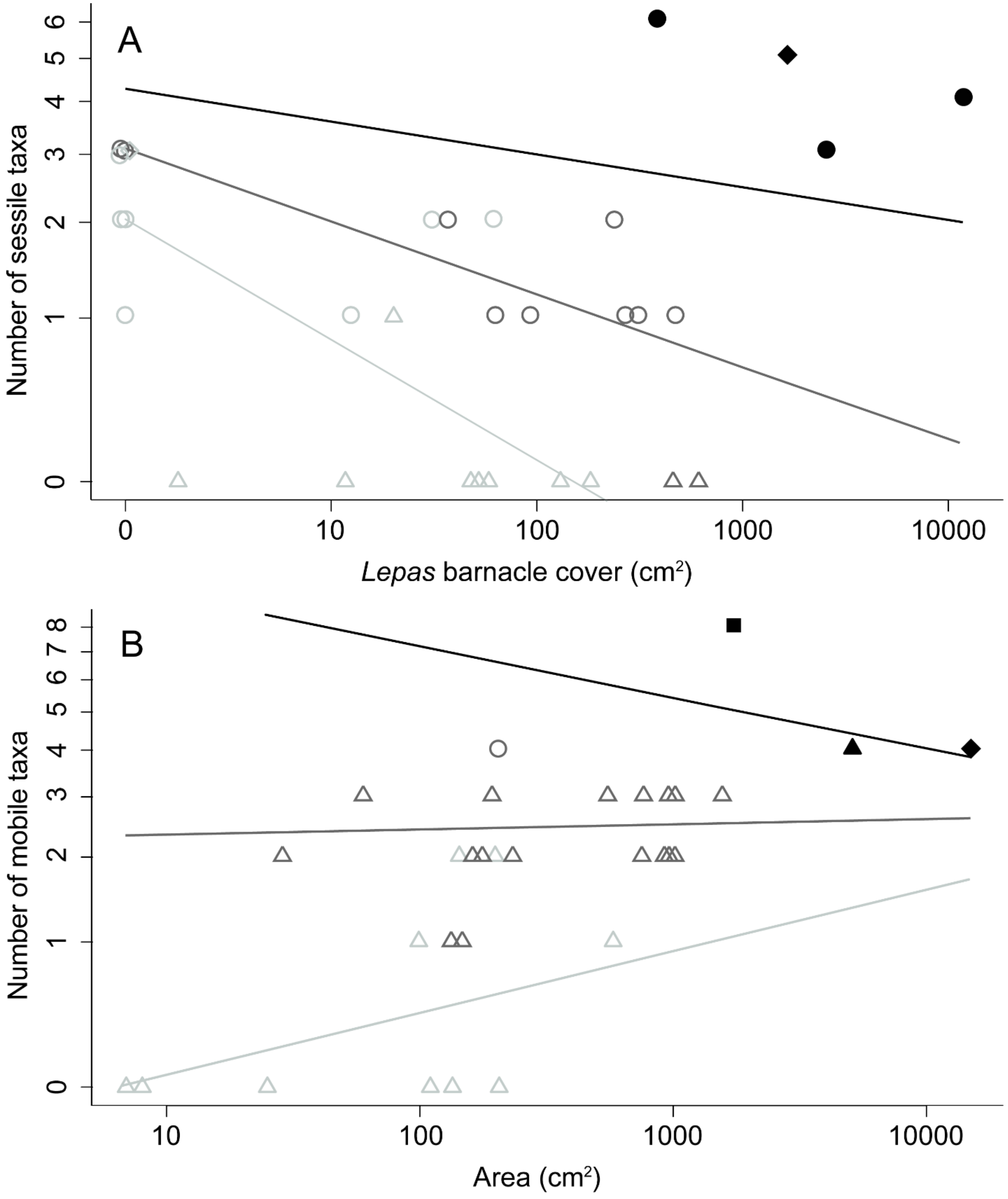

Figure S1: The relationship between A) the areal cover of adult *Lepas* barnacles and the number of sessile taxa; and B) submerged surface area of plastic debris and the number of mobile taxa on the log-log scale. In each plot, data points represent each of the individual pieces of plastic debris

sampled, which included: taxa primarily documented on oceanic rafts (a), taxa commonly documented in coastal habitats (b), coastal taxa not previously documented on oceanic rafts (c), and coastal taxa from the East and West Pacific Ocean (d). The shape of each data point designates the type(s) of sessile (for panel A) or mobile (for panel B) taxa found on the debris piece: triangle, a; circle, a+b; diamond, a+b+c; or square, a+b+c+d. To visualize the interaction between predictor variables in each plot, we separated debris into three groups: low (light grey, open points), intermediate (dark grey, open points), and high (black, solid points) values of the second predictor variable (submerged surface area for A and barnacle cover for B). Lines represent predictions of the best-fit model (interaction model; sessile taxa: $R^2 = 0.29$, $p = 0.0068$; mobile taxa: $R^2 = 0.76$, $p < 0.0001$) for the averages of the data points with low (light grey line), intermediate (dark grey line), or high (black line) values of the second predictor variable. Overlapping points (at 0 barnacles in A) were jittered for visual clarity.
